# Nanocarbon condensation in detonation

**DOI:** 10.1038/srep42151

**Published:** 2017-02-08

**Authors:** Sorin Bastea

**Affiliations:** 1 Lawrence Livermore National Laboratory, Energetic Materials Center, 7000 East Avenue, Livermore, CA 94550, USA

## Abstract

We analyze the definition of the Gibbs free energy of a nanoparticle in a reactive fluid environment, and propose an approach for predicting the size of carbon nanoparticles produced by the detonation of carbon-rich explosives that regards their condensation as a nucleation process and takes into account absolute entropy effects of the cluster population. The results are consistent with experimental observations and indicate that such entropy considerations are important for determining chemical equilibrium states in energetic materials that contain an excess of carbon. The analysis may be useful for other applications that deal with the nucleation of nanoparticles under reactive conditions.

The detonation of common high explosives generates nanodiamonds^1^. This surprising fact has been known for more than a half century and continues to be exploited as a major avenue for producing nanodiamonds for a variety of industrial, medical and bioengineering applications^2^,^3^,^4^,^5^,^6^. Detonation nanodiamonds have been thoroughly characterized and studied, and found to be very suitable for a wide range of novel uses due to their small (typically 4–5 *nm*) and uniform size^2^,^3^,^7^. Yet the condensation process leading to the formation of carbon nanoparticles in the detonation wave of explosives^1^,^8^ remains little understood, and qualitative arguments alone are generally used to rationalize the experimental observations^1^,^4^. Classical detonation science texts^9^ mention the condensation of carbon in negative oxygen balance explosives (i.e. explosives that do not contain enough oxygen to turn all carbon into *CO*_2_ and all hydrogen into *H*_2_*O*) only in passing, but the current consensus is that it plays an important role in determining many of their properties, particularly the energy release characteristics and possibly failure behavior and sensitivity^10^,^11^,^12^,^13^,^14^,^15^,^16^,^17^,^18^. This has spurred renewed interest in this major detonation phenomenon^19^,^20^,^21^.

The appearance of the condensed carbon phase in the detonation products of explosives poses a challenge for the canonical theory of the plane wave steady detonation process^9^,^22^, which envisions a reaction zone extending (in the reference frame of the moving detonation wave) from the von Neumann spike, corresponding to the shocked unreacted material, to the Chapman-Jouguet (C-J) point, residing on the chemically equilibrated shock Hugoniot of the system. On the one hand the carbon nanoparticles recovered from detonations are obviously quite different from the bulk carbon that would necessarily correspond to the full chemical and physical equilibrium postulated at the C-J state. On the other, the evidence for carbon-rich explosives is that they do reach C-J type behavior at charge diameters of a few inches, and the steady state reaction zone does not increase indefinitely with the charge size^23^. Shaw and Johnson^11^ noted that given their small size the surface energy of carbon nanoparticles is considerable and needs to be taken into account when calculating the energy output of an explosive. Their primary, practical concern was with the slow release of this energy through the diffusion-limited coagulation of clusters and progress of the condensed carbon phase towards the bulk state. Viecelli *et al*.^13^ concluded that the surface energy of the carbon clusters is an important contribution to their chemical potential, and generated carbon phase diagrams for particle dimensions of a few nanometers. These size dependent phase diagrams exhibit phase transition lines that are significantly different from those of bulk carbon; such size effects on the phase properties of isolated nanoclusters are well known and confirmed experimentally for many materials^24^. Viecelli *et al*. also implicitly assumed that chemical equilibrium at the C-J state is reached not with bulk carbon, but with these small carbon nuclei. Their successful comparison of calculations based on chemical equilibrium modeling^25^,^26^ with experimental data for the detonation velocity of carbon-rich explosives such as trinitrotoluene (TNT) and the shock Hugoniots of various hydrocarbons, using the size of the carbon particles as an empirical input parameter, provided support for this idea. Nevertheless, no quantitative argument explaining the size of the experimentally observed nanoparticles was advanced or is currently available. This is the primary goal of the present contribution.

## Results and Discussion

The starting point of the analysis is the Gibbs free energy of a condensed carbon cluster containing *n* atoms, which we denote by *μ*^(*n*)^ (*P, T*); we will assume in the following that these clusters can be approximated as spherical. (To simplify the notation, we will leave the pressure and temperature dependence implicit; also, for the time being, we do not specify the phase of the cluster, which could be either diamond, graphite, or liquid.) Viecelli *et al*.^13^ considered bulk and surface contributions to *μ*^(*n*)^,


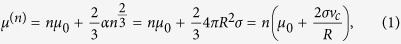


where *μ*_0_ is the chemical potential of the bulk condensed phase, *R* is the cluster radius, *σ* is the surface tension of the phase, with 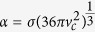, and *v*_*c*_ is the volume per atom of the bulk phase. The above cluster Gibbs free energy yields for an individual nanoparticle the melting point change (with respect to the bulk phase) that is derived using standard assumptions on the applicability of the Laplace law 
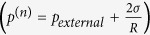
 to the interior pressure, *p*^(*n*)^, of a cluster^27^. Variations of this model remain in current use for the modeling of nanocarbons^28^,^29^. The same approach is also used extensively to calculate the melting properties of metallic nanoparticles^30^,^31^,^32^.

The carbon clusters that occur during the detonation of explosives are produced from the small molecular fragments resulting after the shock-induced exothermic break-up of large metastable organic molecules, and are immersed in a hot, dense, reactive fluid phase containing products such as *CO*_2_, *CO, N*_2_, *H*_2_*O, CH*_4_, etc.^9^,^25^ and likely ionic species^33^,^34^. Mixing and chemical reactions in this multi-component multi-phase system take place with high rates at the high pressures and temperatures typical of detonation, and advance the system towards its chemical equilibrium state. The appearance, dynamics and evolution of the carbon clusters in this complex environment are likely akin to a nucleation process, and cannot be fully evaluated by analyzing only the properties of an isolated carbon nanoparticle. The question of the Gibbs free energy of a small condensed cluster occurring in a fluid phase was originally discussed in the context of the classical homogeneous nucleation theory^35^,^36^, with the goal of determining the equilibrium concentration of clusters and the nucleation rate. Frenkel’s prescription for *μ*^(*n*)^ as given in ref. 35 is


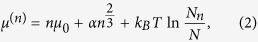


where *N*_*n*_ is the number of clusters of size *n* and *N* is the total number of particles (molecules and clusters) in the mother phase. Thus *μ*^(*n*)^ contains the standard surface energy contribution in the capillary approximation (where the planar surface tension is used for the properties of the cluster), along with the ideal mixing entropy^37^. Lothe and Pound^38^ argued that quantum contributions to the absolute entropy of condensed clusters moving through a fluid phase also need to be considered, the most important of these being due to their translational and rotational degrees of freedom. This fundamental conceptual problem is of continuing interest for the understanding and application of the classical nucleation theory^39^,^40^,^41^. Currently, it is accepted that the rotational contribution is already included in the capillary approximation for the surface free energy^40^,^42^. In the following we will therefore include only the translational effect, using the standard form originally considered by Lothe and Pound for a dilute population of clusters^38^,^43^. Consequently, we write for the Gibbs free energy of a carbon cluster of size *n* immersed in a fluid matrix


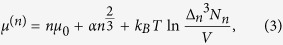


with 
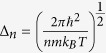
, *N*_*n*_ the number of clusters of size *n, V* the system volume, *T* the temperature and *m* the carbon atomic mass. In the case of detonation the fluid matrix is a reacting mixture, which at the C-J point should reach chemical equilibrium both within itself, and with the condensed carbon phase. If we denote by *μ*_*C*_ the chemical potential of free carbon atoms in the fluid, chemical equilibrium between the mixture and the carbon clusters requires


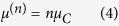


Chemistry in the fluid also involves reactions such as *C* + *CO*_2_ = 2*CO, CO* + *H*_2_*O* = *CO*_2_ + *H*_2_, etc., with corresponding chemical equilibrium equations 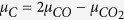, 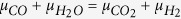, etc. Eq. 4 indicates equilibrium with respect to the transformation of a cluster into *n* carbon atoms. We now assume that individual carbon clusters are also in (unstable) chemical equilibrium with respect to evaporation and condensation of single carbon atoms, i.e. that they are critical nuclei. The applicable classical Gibbs condition^35^,^44^ is 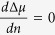, where 
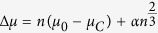
 is the chemical potential difference between carbon atoms in a nucleus and in the fluid. This yields 
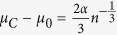
, or using Eq. 4


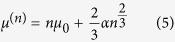


Eq. 5 is identical with the Gibbs free energy previously adopted for an individual cluster, Eq. 1, but as opposed to that relation, refers only to clusters of critical size, as defined by Eqs 3, 4, 5 and appropriate chemical equilibrium conditions in the fluid phase. These equations taken together define both the chemical equilibrium state and the size of the carbon nanoparticles, and in principle can be integrated into thermochemical predictions of detonation or shock properties at high pressures and temperatures^25^,^26^. Here we obtain instead estimates of the size of the carbon nanoparticles generated in detonations based on the thermodynamic conditions at the C-J point and the amount of condensed carbon that is likely to be produced there. To this end we rewrite Eq. 3 as


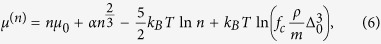


where *ρ* is the system mass density, *f*_*c*_ is the mass fraction of condensed carbon and 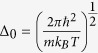. Substitution into Eq. 5 yields an equation for the cluster size *n*


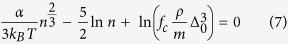


In the following we perform cluster size estimates for a set of five carbon-rich explosives: COMP-B, a mixture of 40% TNT (trinitrotoluene – *C*_7_*H*_5_*N*_3_*O*_6_) and 60% RDX (cyclotrimethylenetrinitramine – *C*_3_*H*_6_*N*_6_*O*_6_) that is the explosive of choice for producing nanodiamonds^4^,^8^, TNT, HNS (hexanitrostilbene – *C*_14_*H*_6_*N*_6_*O*_12_), TATB (triaminotrinitrobenzene – *C*_6_*H*_6_*N*_6_*O*_6_), and BTF (benzotrifuroxan – *C*_6_*N*_6_*O*_6_). The calculations require the density *ρ* and temperature *T* of the C-J state of these materials, along with the mass fraction of the condensed carbon, *f*_*c*_. In principle these can be approximately determined using chemical equilibrium modeling of the detonation products^25^,^26^. Here we use instead literature values for *ρ* and *T*, including calculations and experiments^1^,^45^,^46^,^47^,^48^,^49^,^50^,^51^,^52^, and approximate the mass fraction *f*_*c*_ by *half* of the excess carbon over the oxygen balanced stoichiometry, e.g., *C*_7_*H*_5_*N*_3_*O*_6_(*TNT*) → 1.5*N*_2_ + 2.5*H*_2_*O* + 1.75*CO*_2_ + 5.25*C, f*_*c*_ ≃ 0.14. This is in fact roughly the amount of condensed carbon recovered in enclosed detonations^8^,^53^,^54^. The cluster size estimates are robust with respect to fairly sizable variations of *ρ* and *f*_*c*_, due to their logarithmic contribution to Eq. 7. The temperature has a slightly more pronounced effect, and its exact value is also less certain; we therefore performed calculations for a range of temperatures encompassing the published predictions and experiments^46^,^47^,^48^,^49^,^50^,^51^,^52^,^55^: 2800 *K*–4000 *K* for COMP-B, 2800 *K*–3800 *K* for TNT, 3100 *K*–4000 *K* for HNS, 1900 *K*–3000 *K* for TATB, and 4100 *K*–5700 *K* for BTF. We also estimate that changes of order 20% in the value of the surface energy coefficient *α* result in cluster size variations of approximately 10%. Independent calculations (using Eq. 7) for the three carbon phases yield cluster sizes of ≃10–20 atoms for diamond and graphite clusters, and of order 10000 atoms (≃5 *nm*) (see Table 1) for the liquid clusters. The actual nucleation of carbon clusters in the detonation products of explosives likely involves an interplay and competition between clusters of different phases and sizes. Such effects have been studied for example for crystal nucleation in simple liquids^56^. The calculational framework outlined above for a single carbon phase is easily extended to multiple phases, in which case the size and mass fraction of diamond, graphite and liquid clusters is determined by the following set of equations:


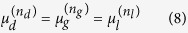



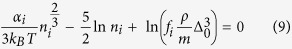



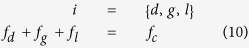


Here {*d, g, l*} stands for diamond, graphite and liquid, *μ*_*i*_^(*n*_i_)^ are chemical potentials defined from Eq. 5 for each of the phases and we employ the same surface energy coefficients *α* as in ref. 13 (incidentally, *α*_*l*_ is consistent with recent measurements for the surface energy of amorphous carbon^57^). The solution of these equations will yield the cluster sizes *n*_*d*_, *n*_*g*_ and *n*_*l*_, as well as the corresponding mass fractions *f*_*d*_, *f*_*g*_, and *f*_*l*_. It requires however the bulk chemical potentials for the three phases, *μ*_0*d*_, *μ*_0*g*_ and *μ*_0*l*_, at the C-J point pressure and temperature. In the following we use the values quoted in ref. 46 for the C-J pressure, while for the bulk chemical potentials we employ both the model of ref. 13 and that of ref. 58; they yield consistent results. For all the explosives studied we find that the diamond and graphite mass fractions are largely negligible, i.e. most of the carbon is in liquid clusters (a possible exception is HNS, where graphite clusters are roughly 1% of the condensed carbon). The size of the liquid clusters is essentially the same as the one found when the liquid is considered alone, i.e. Eq. 7. We show in Table 1 the number of carbon atoms in the liquid clusters and their diameter 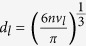, along with the average size of nanodiamonds recovered from experiments^7^,^8^,^59^, or condensed carbon clusters observed immediately after detonation using small-angle X-ray scattering (SAXS) experiments^19^,^60^.

The agreement is reasonable for the first four explosives, with notable disagreement for the last one, BTF. We expect that the liquid carbon clusters undergo rapid quenching from the C-J state due to the volume expansion and concurrent temperature decrease occurring behind the detonation front. This should lead to cluster crystallization to diamond or graphite, depending on the C-J pressure and temperature and the thermodynamic states being traversed by the expansion path. Thus, due to its high detonation pressure COMP-B would be expected for example to yield after expansion a large fraction of nanodiamonds^8^, while TNT and HNS may lead primarily to the production of graphitic clusters^8^,^19^. TATB is an interesting case, with an apparent high detonation pressure, which should favor diamond formation on expansion from the C-J point, but possibly with an unusually low detonation temperature^46^, which may inhibit the crystallization process and could result in structures with more amorphous character. The results reported here suggest that sizeable carbon nuclei are already present at the C-J point. Although their evolution during release remains to be fully elucidated, it will likely include diffusive aggregation on time scales up to microseconds^11^,^16^,^61^. Indeed, the nanodiamonds recovered from detonations are found to be part of larger aggregates that need to be broken up to separate the individual nanoparticles^4^,^7^. For explosives with high detonation temperatures such as BTF the aggregation process may proceed for a longer time in the liquid phase, before crystallization occurs, which may explain the larger nanodiamonds recovered from its detonation^59^.

The above analysis is not only applicable to detonation, but also to strong shock waves propagating through an explosive^62^ or a carbon-rich material^63^. Shock compression of COMP-B to twice its C-J pressure^62^ should yield for example nanoparticles that are least 10–20% larger than those produced in detonation. In the case of liquid CO^63^, shock compression to 20 GPa for example should produce nanoparticles of ≃7 *nm*, while pressures of 40 GPa will likely yield nanoparticles of ≃10 *nm* and possibly larger due to the high shock temperatures reached. These predictions can be tested experimentally.

In summary, we analyzed the definition of the Gibbs free energy of a nanoparticle in a reactive fluid environment and proposed a framework for predicting the size and potentially the phase of carbon nanoparticles produced by the detonation of carbon-rich explosives. The approach regards the condensation of carbon as essentially a nucleation process in a reactive fluid environment and takes into account absolute entropy effects of the cluster population. The results are consistent with experimental observations and indicate that such entropy considerations are important for determining chemical equilibrium states in energetic materials that contain an excess of carbon. They also suggest experimental avenues for controlling the size of the carbon nanoparticles by manipulating the composition of the initial mixture and the applied shock conditions. The method outlined here makes possible thermochemical calculations that self-consistently determine the size of the condensed carbon nanoparticles produced in detonation, which may yield more accurate predictions than simply using it as an empirical parameter^13^. We note that although the present treatment considers only spherical particles, it may be possible to extend it to other particle shapes, for example by using the thermodynamic approach of ref. 64. Finally, it is worth mentioning that the above analysis may also be useful for other applications where the nucleation of nanoparticles in a reactive fluid environment is important^65^,^66^,^67^.

## Additional Information

**How to cite this article:** Bastea, S. Nanocarbon condensation in detonation. *Sci. Rep.* **7**, 42151; doi: 10.1038/srep42151 (2017).

**Publisher's note:** Springer Nature remains neutral with regard to jurisdictional claims in published maps and institutional affiliations.

## Figures and Tables

**Table 1:** Calculated (n_*l*_ - number of atoms, d_*l*_ - diameter) and observed (d_*exp*_ - diameter) carbon cluster sizes.

Explosive	n_*l*_	d_*l*_ (nm)	d_*exp*_ (nm)
COMP-B	7000–14000	4.6–5.6	4.4–5.5^a^,^b^
TNT	7000–12000	4.5–5.4	4.9^b^
HNS	9000–13000	4.8–5.6	5.4^c^
TATB	3500–8000	3.6–4.7	2–3^d^
BTF	14000–25000	5.7–6.9	25–30^e^

^a^Ref. 7.

^b^Ref. 8.

^c^Ref. 19.

^d^Ref. 60.

^e^Ref. 59.
